# Sensing Using Light: A Key Area of Sensors

**DOI:** 10.3390/s21196562

**Published:** 2021-09-30

**Authors:** José Miguel López-Higuera

**Affiliations:** 1 Photonics Engineering Group, University of Cantabria, 39005 Santander, Spain; lopezhjm@unican.es; 2 Instituto de Investigación Sanitaria Valdecilla (IDIVAL), 39011 Santander, Spain; 3 CIBER-BBN, Instituto de Salud Carlos III, 28029 Madrid, Spain

**Keywords:** photonic sensor, optical sensor, optical fiber sensor, light-based sensor, optical transducer, optical channel, optoelectronic unit, interrogation unit, sensor system, smart sensor

## Abstract

This invited featured paper offers a Doctrinal Conception of sensing using Light (SuL) as an “umbrella” in which any sensing approach using Light Sciences and Technologies can be easily included. The key requirements of a sensing system will be quickly introduced by using a bottom-up methodology. Thanks to this, it will be possible to get a general conception of a sensor using Light techniques and know some related issues, such as its main constituted parts and types. The case in which smartness is conferred to the device is also considered. A quick “flight” over 10 significant cases using different principles, techniques, and technologies to detect diverse measurands in various sector applications is offered to illustrate this general concept. After reading this paper, any sensing approach using Light Sciences and Technologies may be easily included under the umbrella: *sensing using Light* or *photonic sensors* (PS).

## 1. Introduction

Driven by the enormous advancement of technology, the world and its organizations have experienced significant changes running, in the current times, towards a society 5.0. The fourth industrial revolution or Industry 4.0 (I4.0) is understood, in general terms, as any systematic work or labor to do business, trade, undertaking, manufacture, services, handicraft, etc., in a particular field, which will improve productivity and outputs, customization for flexible manufacturing, worker safety and security, remote access, access to data across the supply chain for better decision making, and remote and predictive maintenance, among other factors [1].

A considerable amount of specific data (Big Data), after being treated with artificial intelligence (AI), can offer decisions and take appropriate actions without any human activity, in many cases. All aspects of these smart organizations will be interconnected, supported by AI and Smart Devices (SD). Among the essential elements to build up these devices, sensors play a crucial role.

Photonics is considered a Key Enabling Technology (KET) or an Essential Technology for the development of related sciences in Europe, the USA, and other leading nations around the world [2]. The Photonics field, or Science and Technology of Light, is understood as the set of techniques and scientific knowledge which are applied to the generation, propagation, control, amplification, detection, storage and processing of signals of the optical spectrum, along with their technologies and derived uses. Areas such as sensing, communications, advanced manufacturing, and augmented reality, among others, are some of the many areas of photonics. Photonic sensors is, without any doubt, one of its key areas and is also a key subarea of sensors, one that includes all kinds of sensors.

It is worth mentioning that the Photonics market was USD 593.7 billion in 2020 and is projected to reach USD 837.8 billion by 2025, at a Compound Annual Growing Rate (CAGR) of 7.1% between 2020 to 2025. The photonic sensors market was valued at USD 13.25 billion in 2020 and is expected to reach USD 26.65 billion by 2026, at a CAGR of 16.93% over the forecasted period (2021–2026) [3]. Thus, an enormous market potential for the relevant area of Photonics is predicted. Specifically, photonic sensors or sensing using Light, which can be understood as any sensing approach that employs Light-based technologies, is an area with a solid expected socio-economic impact for the first decades of this 21st century.

All those who have been working for a long time ago in sensing, in general, and in photonic sensing, in particular, are submitted to a vast “soup”, or terms that seem to show different meanings which in reality are the same, or are incorrectly used inducing errors for others, or, in chats, papers, or meetings, are speaking about sensors when in fact are talking about transducers, etc.

Can terms, concepts, methods, effects, approaches, techniques, technologies, etc., so disparate, as mentioned in [4,5,6,7,8,9,10,11,12,13,14,15,16,17,18,19,20,21,22,23,24,25,26,27,28,29,30,31,32,33,34,35,36,37], all be considered under the same sensing umbrella?

This invited feature paper offers a comprehensive proposal that aims to clarify the use of the mentioned terms, and many others, and offer an “umbrella” under which all the disparate terms, concepts, methods, technologies, etc., can be easily included. From the accumulated experience from over 30 years working in R&D&I in this area, with this proposal, all sensing approaches using Light science and technology can be very “harmonically” considered inside the sensing using Light area or, simply, photonic sensors.

Independently of any previous background, any reader should be able to easily follow and understand the proposal. The paper is organized as follows: First, terms such as photonics and Light will be clarified; then a conception of sensors and, overall, Photonic Sensors is stated and developed; then, 10 significant cases using different methods, techniques, and technologies to detect different measurands, and placed on several application sectors are briefly commented on to illustrate and clarify the author’ proposal.

## 2. Photonics? Light?

At the end of the last century, the field of knowledge known as ‘Optics’ become inappropriate to accommodate the continuing appearance of new concepts, techniques, technologies, devices, systems, applications and, in general, areas or disciplines based on electromagnetic radiations or flux of photons that range, at least, from the deep ultraviolet to the very far of the infrared radiations. X-rays and Gamma rays can also be considered as a flux of photons. For these reasons, as happened with Electronics, it seemed logical to rename the field into a new and more inclusive one, and it was done. Thus was born the Photonics field, or the Science and Technologies of Light [38].

***Photonics** is understood as the comprehensive or inclusive field of knowledge that includes the devices, technologies, techniques, methods, and scientific knowledge applied to the generation, propagation, control, amplification, detection, storage, processing, etc. of Light signals and derived uses.*

With this modern conception, in short: (i) light is considered any electromagnetic radiation or flux of photons of frequencies inside the optical spectra that ranges from the gamma radiations (at least from the vacuum ultraviolet—100 nm) to the very far infrared coexisting with the microwave field inside the terahertz band; (ii) Photonics is the inclusive field which is “doing things” using light science and technology.

Electronics and Photonics are key fields for developing science, technology, and all kinds of organizations, with significant economic and societal impacts. Today, Photonics will represent for the 21st century what Electronics meant for the second half of the 20th century. Both fields coexist in ‘complementary harmony’ and, in many cases, overlap [39].

The Photonics field can be divided into several areas, with **sensing using Light** or **Photonic Sensing** being a very relevant area of the Photonics field breakdown [39,40].

## 3. Photonic Sensors

### 3.1. Sensors

The detection, capture, measurement, supervision, and control of magnitudes of given objects are requirements of paramount importance in today’s times. Getting specific information about a given parameter or measurand (Mx) in any state or domain on/inside an object (Oy) placed in a given environment is commonly recognized as a detection process. *Sensors are the devices or systems developed to detect and capture physical, chemical, biological, and biomedical measurands, and translate and reproduce them in the electrical domain to be useful in real applications in today’s world*.

#### Photonic Sensors

*Photonic sensors (PS) are the devices/systems designed to carry out the faithful reproduction of the measurand in the domain using photonic technologies in its key sensor parts [38].*

**Figure 1:**
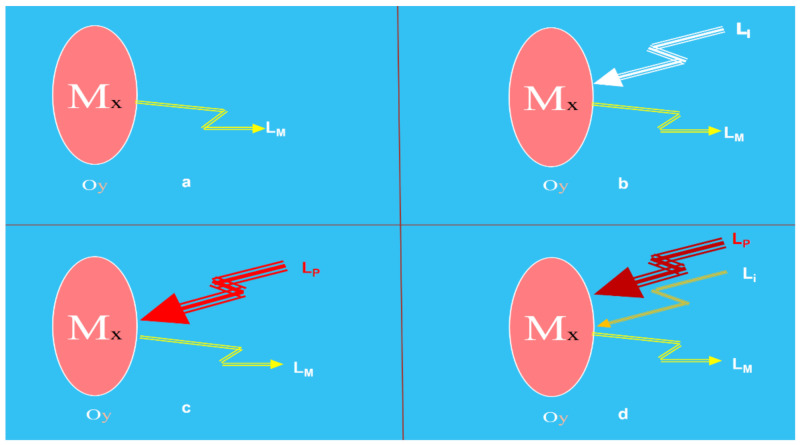
The focus at the measurand on/in the object illustrates main sensing possibilities using light techniques. Light modulated by the measurand (L_M_) on/in the object: (**a**) produced by the object itself; (**b**) returning from the object consequence of the interrogation light (Li); (**c**) produced as a consequence of a pumping light (Lp) and (**d**) produced as a consequence of a pumping and interrogation lights. Courtesy of the author.

In any photonic sensor, the Light (Lx) coming from the object (Oy) includes the information concerning their specific measurand (Mx) that, after being detected and processed, enable their faithful reproduction in the electrical domain. The photons of the Light, Lx, from the Oy can be produced by the object itself or could be a consequence of their excitation with appropriate optical radiation or any other source of excitation energy (Figure 1). The Light from the target includes information (modulated by the measurand or modulating signal) in several of its main characteristics such as amplitude, phase, frequency, polarization, or any other Light aspect [39,40].

**Figure 2:**
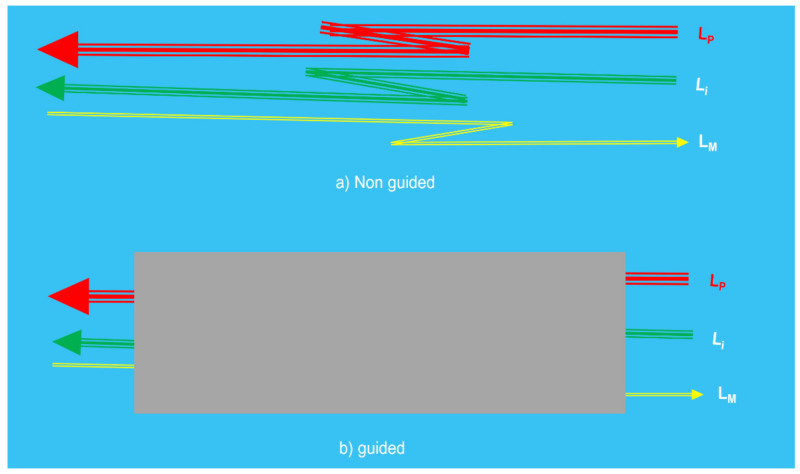
Illustration of the optical channel of a photonic sensor. According to the media used to transmit the Light to and from the object: (**a**) non guided optical channel and (**b**) waveguide optical channel (typically optical fiber cables). Courtesy of the author.

The optical radiations or lights to and from the object can be transmitted by using or not using waveguides. Then, another part of the sensing device is induced: the optical channel (Figure 2).

**Figure 3:**
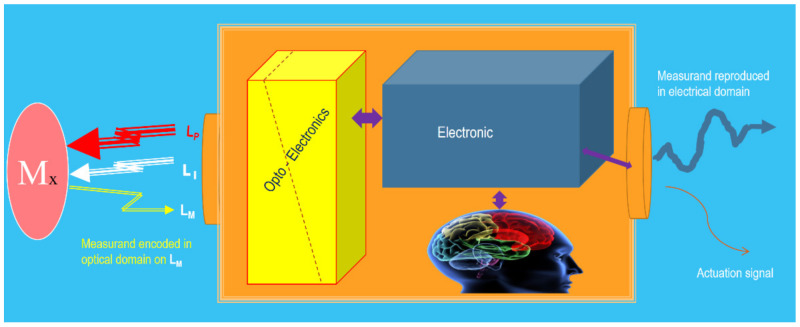
Illustration of the optoelectronic unit of a photonic sensor. It is in charge of producing and controlling the photonic beams or Light (if required) to carry out the object’s interrogation or/and pumping tasks. It is also in charge of achieving the photodetection, and all required additional processes, to reproduce the measurand in the electrical domain. When equipped with intelligence capable of giving an actuation signal, the sensing device is transformed into a Smart Photonic Sensor (SPS). Courtesy of the author.

On the other hand, another essential part of an actual photonic sensor is the optoelectronic unit (OU). This unit is in charge of generating the appropriate lights (if required) to carry out the object’s interrogation or/and pumping tasks. It is also in charge of achieving adequate photodetection, pre-amplification, demodulation, and additional (if required) tasks such as equalization, digitalizing, processing, etc., to reproduce, with the pre-specified quality, the measurand in the electrical domain (Figure 3).

When the optoelectronic unit is equipped with some kind of intelligence, in addition to the sensed signal or, instead, the sensing system is capable of giving an actuation signal, the sensing device is transformed into a Smart Photonic Sensor (SPS) [41,42,43]. Thus, an SPS can be understood as the photonic sensor system that includes smartness capable of offering actuation signals to allow appropriate reactions or interventions on/in the object (Oy) from which the Lx modulated Light is coming. The mentioned intelligence, commonly placed in the optoelectronic unit, is created using programs running specific algorithms.

**Figure 4:**
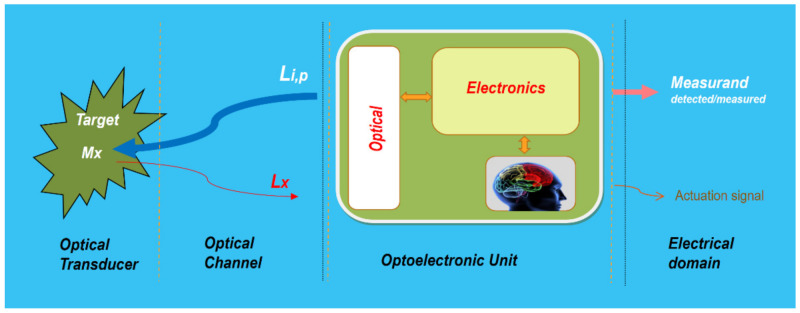
Illustration of the photonic sensor concept and its block diagram. The photonic sensor provides representative and faithful electrical signals of the measurands on/in a given object or target. When a PS is equipped with intelligence and provides actuation signals, it is transformed into a Smart Photonic Sensor (SPS). It is integrated into three main parts: optical transducer, optical channel, and optoelectronic unit. Courtesy of the author.

According to everything mentioned above, a photonic sensor is integrated or constituted, in general terms, by three main parts or blocks: the optical transducer, the optical channel, and the optoelectronic unit (Figure 4). The optical transducer is the part in which the measurand modulates the Light; the optical channel is in charge of the optical connections between the transducer and the optoelectronic unit; the latter (OU), is the part in which the optical signal coming from the object (L_M_) is photodetected, amplified, demodulated, processed, equalized, etc., offering as a result an output electric signal (analogic or digital) which is a faithful reproduction of the measurand [38,41,42,43]. The optoelectronic unit also includes, if required, all technology concerning the optical source(s) to interrogate and/or pump the object to induce the appropriate light response (L_M_).

**Figure 5:**
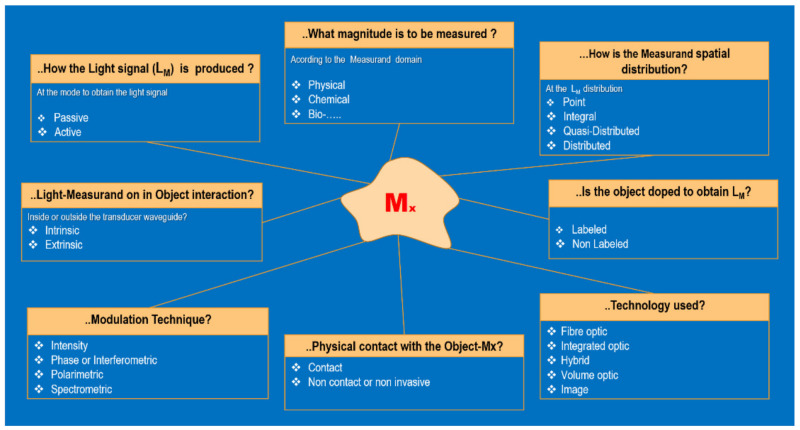
Types of photonic sensors taking into consideration aspects concerning the measurand (Mx) on/in the object (Oy) and the modulated Light (LM). Courtesy of the author.

As shown in Figure 5, considering aspects concerning the measurand (Mx) and the modulated light (L_M_), different blocks of knowledge to define each photonic sensor can be obtained.

**Figure 6:**
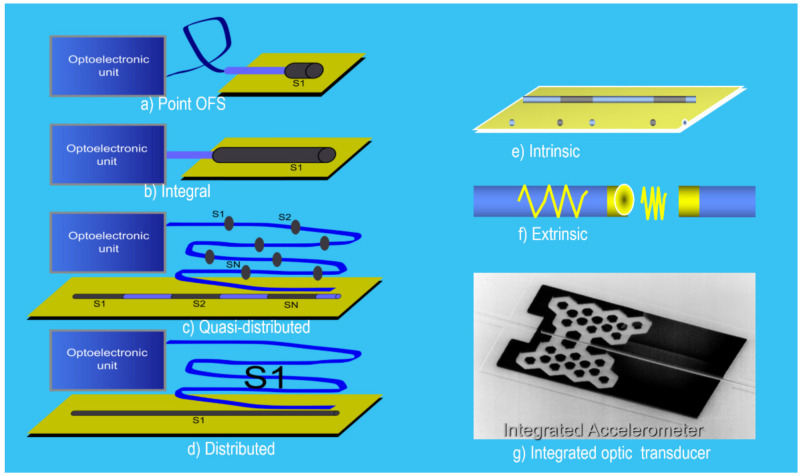
Illustration of photonic sensors according to the technology and the interaction of light-Oy-Mx: (**a**–**f**) Optical Fiber Sensors and (**g**) Integrated Optic Sensor. Courtesy of the author.

Thus, several sensor types can be defined according to:

(a) the modulating technique used to encode the measurand on L_X_. The PS can be of *intensity*, *phase* or *interferometric, polarimetric* and *spectromet*ric when Mx is encoded on the intensity, phase, polarization, or spectrum, respectively, of the Lx signal;
(b) interaction of Light-Object-Measurand. The PS can be *intrinsic* when the interaction is indirect (Light always remains inside a waveguide) or *extrinsic* when the Light interacts directly with the object (Light is not inside a waveguide during the interaction);
(c) way or mode to obtain the L_M_. The PS can be *active* or *passive* when producing the Light requires the pumping or excitation of the object or the waveguides of the transducer (if any) to generate the Lx, respectively;
(d) the measurand’s domain. The PS can be *Physical*, *Chemical*, *Biological*, *Mechanical*, *Biomedical*, etc., when the Mx on/in the object is in physical, chemical, biological, mechanical, or biomedical domains, respectively;
(e) the spatial distribution of the measurand in the object. The PS can be *punctual, integral*, *quasi-distributed,* or *distributed* when the detected measurand is in a point, or is the result of the integral of the measurand along of a given line or an area, or is distributed in separate points along a line, or is fully distributed along a line or surface, given a spatial resolution, respectively;
(f) the requirement to dope or not the object to obtain the Lx. The PS can be *Labeled* or *Non-Labeled*, respectively;
(g) the technology used to build-up the transducer. The photonic sensor can be an *Optical Fibe*r sensor, *Integrated Optic* sensor, *Hybrid* sensor, *Volume Optical* sensor, or *Image* sensor when fiber optic, integrated optic, hybrid, volume optic, or image-based technologies are used, respectively (Figure 6).

To clarify the usefulness of those mentioned above to define, appropriately, a given PS, let us show three examples.

1. The transducer is made by using fiber technology. It measures the strain on N points along a length of fiber placed inside an aircraft wing. The measurand is encoded in the spectrum of an interrogation light signal that is always inside the fiber using Fiber Bragg gratings (FBGs); to interrogate the transducer, pumping energy is not needed. The output of OU gives only a faithful reproduction of the measurand in the electric domain. With this data, an appropriate definition of the PS could be a *Bragg grating Optical Fiber sensor for Quasi-distributed strain monitoring in an aircraft wing*. Or, *Quasi-distributed Optical Fiber Bragg Grating sensor for strain monitoring in an aircraft wing*. It is not necessary to mention that it is passive, neither that it is intrinsic, nor that is spectrometric, because it is deduced from the FBG technology used.
2. By using the excitation with a pulsed light pump source of a surface of an X composite material; without any contact with the object, their emitted infrared radiation is acquired after the pump pulse, properly treated (in the OU), and the sub-surface in-homogeneities (defects) are detected. An adequate definition of this PS could be an *Active Thermographic Non-contact photonic sensor for sub-surface defects detection in X composites*. Here, non-contact can be removed, and extrinsic is not necessary to be mentioned because of the thermographic technology used.
3. By using the appropriate laser illumination of a hand finger and Doppler Effect, in an adequate OU, the speed of the blood inside the finger vessels (and hence the blood perfusion map) is determined. When the perfusion is below a level, an actuation signal is offered by the OU. A possible definition of the PS could be a *Non-Contact Doppler-based Smart Photonic Sensor for monitoring spatial distribution of blood perfusion in hand fingers*. Here, the terms based and spatial can be removed.

## 4. Ten Significant Cases of Photonic Sensors

This conception of photonic sensors or SuL offers an umbrella in which any sensor using Light Sciences and Technologies can be appropriately included. An overview of 10 significant cases using different modulating techniques, employing diverse technologies to detect different measurands placed on/in objects, in different environments and sector applications will be briefly presented in the following lines to demonstrate this vision objectively.

It is worth mentioning that in this vast area of Photonics, many other cases and references could have been used to illustrate the proposal. However, without any reductions of the scientific level or any decrease in the reaching of this section’s primary goal, the author decided to select an illustrative set of examples. The cases included (mainly of his R&D group to avoid any inconvenience with intellectual rights, additional bureaucracy, etc.) are situated on hot topics in sensing using Light and with a high level of interest in the current times.

It must also be worthy of mentioning that this section’s goal is not to do a review of the existing literature that, as said, is vast. Instead, a brief and synthetic overview of each selected case is offered to the reader to increase the usefulness of the paper.

### 4.1. Photonic Sensors Based on Optical Fiber Technology

Photonic sensors based on optical fiber technology, commonly known as Optical Fiber Sensors (OFS), are attractive in cases where they offer superior performance compared with the more proven conventional sensors, and offer in addition: (i) improved quality of the measurements, (ii) better reliability, (iii) the possibility of replacing manual readings and operator judgment with automatic measurements, and (iv) an easier installation and maintenance or a lower lifetime cost [44].

The application areas for OFS are extensive, including civil or industrial structure monitoring (concrete beam tests, bridge girders, ore mines, nuclear containers, tunnels, hydroelectric dams…), or composite materials (spacecraft, aircraft’s tail spars, helicopters, and windmill rotor blades, ship and submarine hulls, composite cure monitoring, composite girders for bridges…). In addition, OFS Technology can also be employed on acoustic sensing (towed hydrophone arrays, down-hole sensors for oil wells) on in-plant or distribution of electric power utilities, for gas pipelines and, in general, for control and monitoring of industrial, medical and even environmental processes [45,46].

An extensive set of points, integral, quasi-distributed, and fully distributed photonic sensors based on optical fiber technology can be found in scientific reports and adequately working in real applications. SOFO approaches and speckle-based fiber sensors are both effective techniques for integral measurements [47,48]. For Point and quasi-distributed measurements, fiber-optic microstructure sensors including Fiber Bragg grating (FBG), long-period fiber grating, Fabry-Pérot interferometer sensors, Mach-Zehnder interferometer sensors, Michelson interferometer sensors, and Sagnac interferometer sensors are widely used [49]. On the other hand, optical fiber distributed sensors, key OFS technology based on linear scattering (Rayleigh) and non-linear scattering (Raman, Brillouin) are the more relevant used techniques [50,51]. Two recent cases, one based on a hybrid configuration to measure very high temperatures and another based on speckle for on-bed patient measurements, will be very briefly reviewed below.

#### 4.1.1. Ultrahigh Temperature Hybrid Fiber Sensor

**Figure 7:**
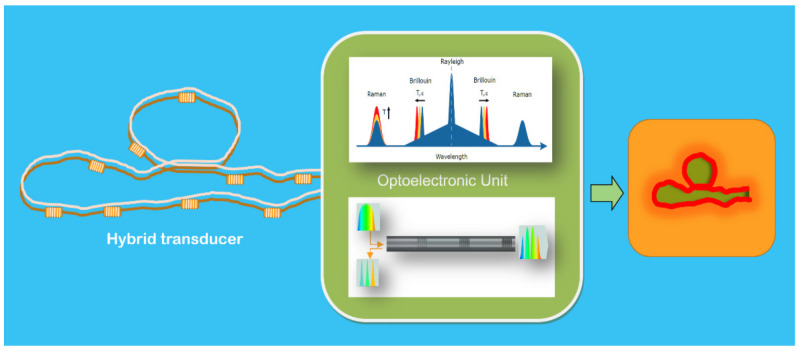
A general schematic illustration of a versatile Hybrid Optical Fiber Sensor for distributed high-temperature industrial processes monitoring. The hybrid transducer is composed of one or two fibers (unsubmitted to any elongation) inside a special cable: (a) when the cable contains only a fiber, a set of Fiber Bragg Gratings (FBGs), able to work correctly at the specified high temperatures, is placed along with transducer fiber; (b) when two fiber are inside the cable then in one of them a set of high-temperature FBGs are placed along with the fiber to quasi-distributed temperature measurements, and the other one is there to do the distributed temperature determination along with the fiber with a given resolution. In both cases, the temperature offered using FBGs is also used to calibrate the distributed measurements. In the OU, Raman Scattering and tunable laser-based sub-sections (in different wavelength rages) interrogate both the distributed and the quasi distributed transducer transducers. The appropriate signal processing enables the determination of the distributed temperature along the length of the transducer cable. The results are presented in a proper synthesized image representative of the spatial high-temperature distribution on/in the object in which the transducer is installed, and (if required) some specific intelligence is added; actuation signals can be offered at the OU’s output. Courtesy of the author.

Fiber Bragg grating (FBG) and Raman-based distributed temperature sensors (RDTS) are to date widely used by many fields of industry for both quasi-distributed and distributed measurements (Figure 7). Furthermore, the combination of these two technologies is of great interest in industrial environments. It allows complete sensing of different structures: distributed temperature measurements and the structure and strain/temperature measurements in areas of particular interest. The distributed sensing does not have the required spatial resolution and exactness. Moreover, it offers cost-effective and low-complexity systems in comparison to other alternatives presented in the associated literature.

The sensor was checked in the field in the facilities of a components manufacturer company for the nuclear industry. Distributed and quasi-distributed temperature measurements were carried out up to 600 °C. In the transducer, pure silica multimode gold-coated fibers and FBGs inscribed in the same multimode fiber were used. The FBGs were fabricated employing the point-by-point technique in a simple setup for Type I femtosecond inscription. Their exceptional resistance to high temperatures and strain up to approximately 4144 𝜇𝜀 was demonstrated. The ROTDR (Raman Optical Time Domain Reflectometry) measurements were calibrated to correct the dynamic variations with the temperature of optical losses in the gold-coated fiber [52,53,54].

#### 4.1.2. Quasidistributed OFS for Structural Integrity Monitoring of Wind Turbine Blades

Humans require a much more sustainable environment, and the current crisis has paved the urgency to find new clean energy sources. However, to harvest renewable energy more efficiently, the size of the present device structures has become physically more significant and complex, making maintenance and repair works difficult. The latter exponentially increases with the extreme severity of the working and remote harsh environments.

During their working lives, due to potential damages or deterioration induced by environmental degradation, wind turbine structures are subjected to adverse changes in their structural health conditions, wear, errors in design and construction, current loads, overloads and some unexpected events like earthquakes or impacts or, simply, by their normal working life [41]. Therefore, it is desirable to assess their structural health conditions to mitigate risks, prevent disasters, and plan maintenance activities optimally. The drives mentioned above require technologies to detect possible degradation/damage over some time, estimate the effects of the external loads, estimate the remaining service life, and make the structures more lightweight, more reliable and cost-efficient. To reach these requirements, it is necessary to include in the blade’s structure a kind of system that can automatically detect the damage, characterize it (recognize, localize, quantify or rate), and report it, providing important data that can be used to optimize the operation, maintenance, repair, and replacment of the structure based on reliable and objective data.

**Figure 8:**
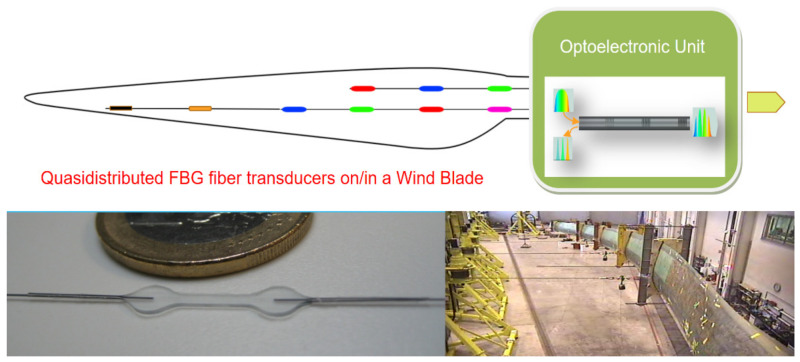
Schematic illustration of a Quasidistributed FBGs Optical Fiber Sensor strain on/in wind blade monitoring. Two fiber transducers are integrated by concatenated Fiber Bragg gratings (FBGs) and interrogate the OU automatically. Below left, a view of the FBG transducer (developed and patented) that is embedded inside the blade structure during its fabrication. Below right is a view of the trials to check the technology up to the blade’s destruction. The appropriate signal processing and algorithmic enables the determination of the quasi distributed strain on/in key places of the blade to reconstruct the more significant strain distribution to know the blade’s structural integrity. Figure made by the author based on material courtesy of the Photonics Engineering Group of UC and Aeroblade.

As electric utility wind turbines increase in size, and correspondingly, increase in initial capital investment cost, there is an increasing need to monitor the structure’s health. Acquiring an early indication of structural or mechanical problems allows operators to better plan for maintenance, possibly operate the machine in a de-rated condition rather than taking the unit off-line, or in the case of an emergency, shut the machine down to avoid further damage. To build an accurate Structural Health Monitoring (SHM) system in the wind turbine, photonic sensors based on fiber technology installed on/in the blades are essential [41,42,43,44,45,46,47,48,49,50,51,52,53,54,55]. Even though several fiber optic transducer technologies are being employed to constitute the “nerve” part of the smart fiber optic sensor system, here, we will only briefly mention one based on FBGs (Figure 8).

Employing FBG based transducers, a wind turbine blade manufactured by the company Aeroblade Inc. has been monitored. A quasi distributed FBG transducer including 15 strain transducers was installed on the blade. The OFS sensor system capable of retrieving the strain distribution of a loaded structure has been experimentally verified during four loading tests in very different loads of the structure. All sensing measuring points have been distributed to critical areas to evaluate the wind turbine blade response. The final tests exhibited outstanding linearity and repeatability, proving the feasibility of applying FBG-based sensing technology in this field of application [56]. The technology is patented [57].

#### 4.1.3. Integral Speckle Fiber Optic Sensor for Patient Monitoring on Bed

With the focus on the health of citizens, care organizations and their professionals require new technologies to enable better and more reliable diagnostics in shorter times to take appropriate decisions. This is of particular interest to elongate the healthy period of older people, for which non-contact, low-cost technologies able to supervise representative parameters of their health are of paramount importance. Therefore, new sensors and smart sensors are required.

In terms of patient monitoring, current sensing methods have some disadvantages, making them inconvenient for continuous vital signs monitoring. For example, the need for sticking sensors to the skin reduces the patient’s mobility and can be uncomfortable. Moreover, sensors need to be replaced each time the patient needs to be moved, even for a short period.

Fiber-optics-based techniques have grown in importance within the non-contact monitoring field due to their versatility and possibility of being used with singular environments, such as magnetic resonance imaging scans. In these environments, placing the metal or standard electronic components is not an option as it can cause them to heat up and malfunction.

Among fiber optics techniques, speckle technology has emerged as a promising method for monitoring heartbeat and motion due to its high sensitivity and relatively low cost. One of the first tests was carried out at the University of Virginia in 2004. It concluded that these types of sensors have the potential to become a cost-effective method of automating long-term monitoring of patients [58]. New R&D works based on this technique have been carried out, contributing to the advance of state of the art [59,60,61]. Since then, the advancement of computing technologies and their mass production have enabled the development of new sensing devices, able to work in real-time and at low cost, which at that time was impossible.

Considering all those mentioned above, a smart, low-cost, speckle-based optical-fiber sensor to detect and measure heart rate (HR) is briefly presented. Furthermore, the proposed devices can measure HR and motion without directly contacting people’s skin, even when lying in different positions [62].

Coherent laser radiation is injected into the core of a multimode fiber. Then speckle is generated by the spread of a large number of modes with different phase velocities. The larger the normalized frequency (V), the higher the number of modes (M) propagating along the waveguide, with different propagation constants. Along with the fiber, the modes that coincide in the waveguide’s same spatial point suffer interference (if the fiber place is inside the coherent length of the optical laser source). Then a transversal speckle pattern is created with the interference’s contribution of all modes. Suppose the fiber length is below the coherent length of the source. In that case, interference effects are well structured and can be observed through the end of the fiber, being that this speckle pattern is extremely sensitive to any change of the optical paths of each mode. This fact supports the conclusion that the speckle in multimode fibers can be used as an integral fiber transducer extremely sensitive to any perturbation on the fiber, being able to detect infinitesimal vibrations and or motions (Mx).

**Figure 9:**
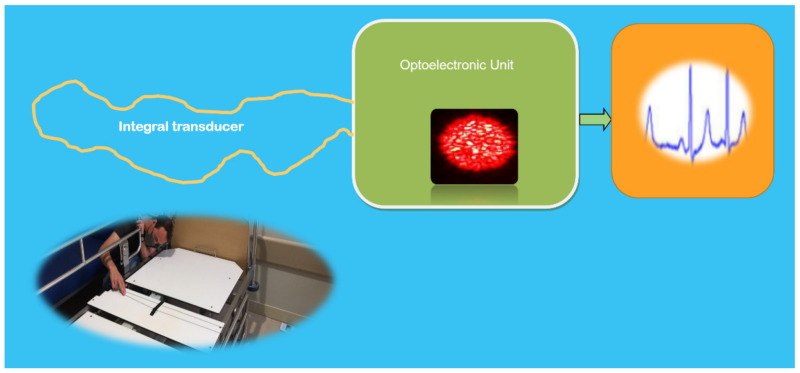
Schematic illustration of an integral specklegram based sensor for non-contact monitoring of vital signs of humans on/in bed. Monochromatic light is injected in the multimode POF fiber with a length lower than the laser coherence length. A low-cost tiny CCD device detects the speckle image at the end of the fiber. The changes in the intensity of successive speckle frames are functioning on the fiber perturbation. Their treatment with appropriate further processing drives to the time-dependent HR signal. In the inset, below left, is shown a view of installing one of these sensors on a bed (below the mattress) the service of Cardiology of the Hospital Universitario Marqués Valdecilla del SCS en Santander, Spain. Courtesy of the author.

With this in mind, to detect vital signs of patients lying on/in bed, an integral speckle-based optical fiber sensor has been conceived, developed, checked in the laboratory, and is being validated in field conditions. As shown in Figure 9, the integral transducer is constituted by an appropriate multimode Polymer Optical Fiber (POF). The semiconductor laser source beam (638-nm wavelength) is launched into the transducer fiber. The speckle pattern at its end is detected by a very tiny, low-cost CCD camera that produces data at an appropriate rate (frames per second). As the changes in the intensity of successive speckle frames are dependent on the fiber perturbation, a one-dimensional and time-dependent intensity change signal that summarizes all the speckle perturbation information is generated. Appropriated further processing converts the intensity of the speckle signal into a time-dependent HR signal. It is worth mentioning that this transformation has to take place in near real-time conditions (inline processing), transforming the perturbation value into HR value and motion detection [62]. Currently, this patented [63] fiber-based photonic sensor developed in the frame of the TeDFES (European funds) is being validated in several services of two hospitals of the SCS in Santander, Spain.

### 4.2. Photonic Sensors on Integrated Optics Technology

BioPhotonic Sensor: Nanophotonic Biosensor for Point-of-Care COVID-19

In general terms, a biophotonic sensor (BioPS) is a photonic device able to detect any substance with high or ultrahigh sensitivity using a specific and selective bio-molecular recognition that is highly desirable to be in real-time and very fast. With photonic biosensors, biospecies such as proteins, DNA, pathogens, virus, bacteria, and toxic pollutants, among others, could be detected and quantified [64,65].

**Figure 10:**
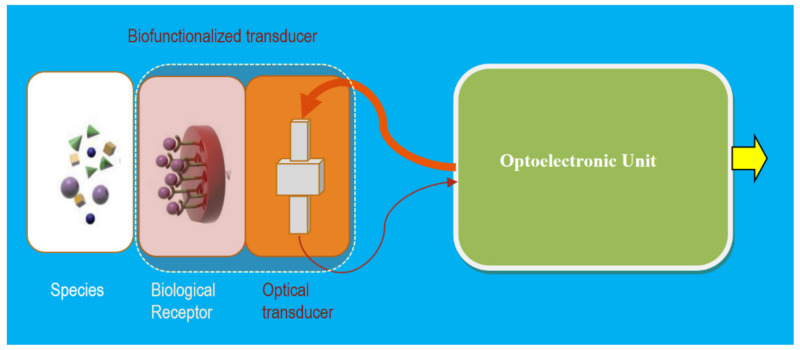
A general schematic illustration of a BioPhotonic Sensor: The bio-functionalized transducer is constituted by a specific biological receptor on the right part of an optical transducer; only the specific bio-species (Mx) will be placed on the particular receptor changing (modulating) the optical properties on the optical transducer response. These changes are detected and correlated with the Mx and, after the proper processing, the output is given by the optoelectronic unit. If some kind of specific intelligence is added, then an actuation signal could be offered at the OU’s output. The optical transducer can be implemented using different technologies, being more valuable than the integrated optics one. Courtesy of the author.

As shown in Figure 10, schematically, a BioPS typically is constituted by a bio-functionalized transducer to capture only the desirable specific bio-species that change (modulate) some property of the returning Light (LM) to the OU. These changes are detected and correlated with the particular bio-measurand, and after the proper processing, a representative output is given by the optoelectronic unit. In these cases, specific smartness is added to the OU that offers an actuation signal as a result. The bio-functionalized transducer is commonly implemented, placed appropriately on the optical transducer. This specific biological receptor only hosts or receives samples of the particular species for the bio-functionalized transducer. The optical fields that interact with the bio-species could be implemented using different methods, such as the relevant evanescent fields generated by plasmonics and optical nanowaveguides.

Therefore, the BioPS is based on plasmonics, microrings or microspheres resonators, photonic crystals, micro/nanofibers, silicon micro/nanowires, and interferometric schemes to reach the required ultrahigh sensitivities that are commonly used [66,67,68,69,70]. The optical transducer can be implemented using different technologies, being the more useful the integrated optics to develop, for instance, point-of-care PoC micro/nano photonic bio-sensors.

In the very promising silicon photonics technology platform, several interferometric schemes are used to build-up the integrated miniaturized transducers to offer ultra-high sensitivities, high multiplexability, and their mass production capacity, with their low cost enabling the production of disposable or single-use transducers.

Point-of-care nanophotonic biosensors are being developed for the direct, fast, and specific identification of specific bio-species of interest for clinical practice. In addition, they can be implemented in decentralized settings to enable early diagnosis and clinical management of patients.

As a relevant and representative case of this technology, it is worth mentioning the nanophotonic biosensors for point-of-care COVID-19 diagnostics and coronavirus surveillance, which are being developed in the CONVAT European Project [71]. The new technology will provide quantitative detection of the viral load to improve early diagnosis and clinical management of patients infected with COVID-19 by introducing a PoC label-free nanophotonic biosensor for the direct, fast, and specific identification of SARS-CoV-2 without requiring complex equipment.

**Figure 11:**
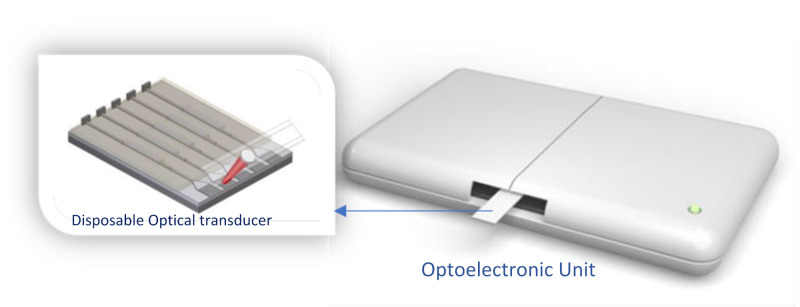
Illustration of the optoelectronic unit and a very ample view of the disposable optical bimodal waveguide (BiMW) interferometric technology considered in the CONVAT project. Made by the author based on material courtesy of Laura Lechuga [64].

CONVAT employs an innovative design of an evanescent-wave nanophotonic sensor based on silicon photonics Bimodal Interferometric technology, BiMW, which has been previously demonstrated for the direct detection of tumor biomarkers and other pathogens with exceptional sensitivities (see Figure 11). Preliminary results were reported on the COVID-19 diagnostic novel approaches and the benefits that PoC nanophotonic biosensors may enable, as envisioned in the project, together with the impact and perspectives for such technology as a boost to global healthcare [71].

### 4.3. Non-Contact Photonic Sensors

#### 4.3.1. Smart Photonic Sensors Based on Infrared Thermography for NDT Applications

An object’s emitted or stimulated infrared radiation can be correlated with the temperature of each fraction of its surface and/or with what is below it. This essential working hypothesis makes it possible to obtain thermographic images and their evolution over time in non-invasive and non-contact ways. The study of the spatial distribution of the surface temperature through treatment with appropriate algorithms and image processing and relating them to the heat energy transfer mechanisms in solids allows, potentially, to obtain three-dimensional information from them and, therefore, enables the detection of defects both in the surface and in the subsurface in a wide range of materials. Infrared thermography (IRT) allows mapping the surface temperature of an object without contact in a remote way by sensing the thermal energy radiated from objects in the infrared band. Smart Photonic Sensing devices based on IRT (SPS-IRT) can be used as a non-destructive test and inspection technology, allowing information to be extracted on the state of its structure or its internal behaviours. They also enable the inspection of large areas in short times with the great advantage of the device’s portability for in-situ inspections.

SPS-IRT can be advantageous for the non-destructive evaluation of materials and structures and monitoring their degradation with time. In addition, these devices can be exploited to find heat losses for energy-saving purposes.

**Figure 12:**
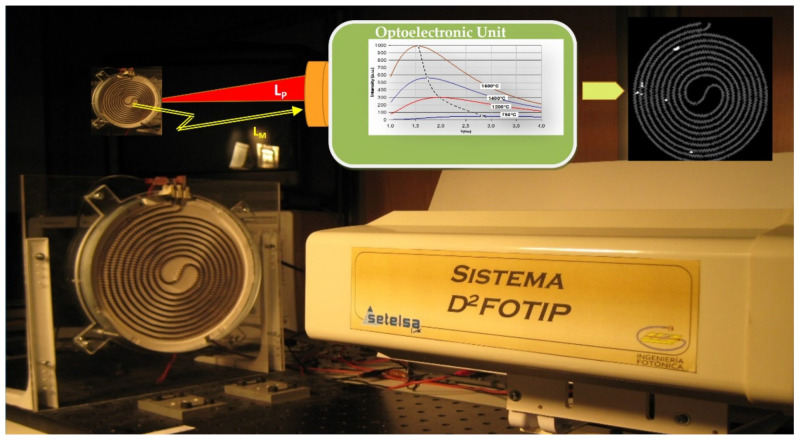
Illustration of SPA-IRT sensor device for online quality control of radiant heaters. After a short energy pulse, the infrared radiations of the cooling process of the heaters were captured and properly treated/processed with adequate algorithms; images with detected defects were synthesized and the OU’s output offered actuation signals. Made by the author based on material courtesy of Photonics Engineering Group of the University of Cantabria, UC.

Just as one case, SPS based on active thermography was developed for defects assessment on radiant heaters. In this particular case, instead of Light pump energy, the short pulse of electrical energy excitation was applied in synchronism with the SPS. This infrared image-based device captures the cooling process of the heaters (Figure 12). After the appropriate processing with the adequate algorithms, the location and classification of defects were achieved. Several defects were considered: lack of supporting brackets; defects originated by a deficiency in the heating material; those from an excess of heating material; and those parts of the heating elements which are in wrong contact (non-contact or semi-buried) with the substrate. Each kind of analyzed defect has a different thermal history after the electrical excitation because of its nature. The defects were represented in an image format in which the defects were spatially located by the optoelectronic unit [72,73]. This SPS-IRT-specific device was developed to perform an online fabrication quality control of vitro-ceramic ovens.

As this general concept of SPS-IRT devices is very promising for an extensive set of sensing tasks, there is in progress a remarkable R&D activity in the subject [73,74,75,76,77]. Applications are numerous, e.g., monitoring mechanical tests, thermo-fluid-dynamics, thermoelastic stress analysis, civil engineering and buildings, aerospace and industrial applications, applications to the cultural heritage, wind turbine blades monitoring, and photovoltaic solar panels monitoring, among others.

#### 4.3.2. Plasma Spectrometric Photonic Sensor for Welding Monitoring

Welding plays a significant role in various industrial scenarios, with relevant examples in the energy sector (pipelines, wind turbines, nuclear generators, among others), aeronautics, the automotive industry, and civil engineering, just to mention some relevant examples. Although there are many different welding processes, from electron beam welding to friction stir welding, arc and laser welding are two of the more significant and popular varieties.

Within this framework, some industrial scenarios exhibit demanding requirements regarding the resulting quality of the seams. However, the complexity of the process mentioned above has made it challenging to obtain theoretical models that offer a suitable performance in process design [77]. Thus, efficient online monitoring is of great interest to allow not only a real-time detection of the appearance of a defect, thus allowing an in-situ repair when possible, but also to correct some parameters during the process in an attempt to prevent or reduce the adverse effects of those flaws.

**Figure 13:**
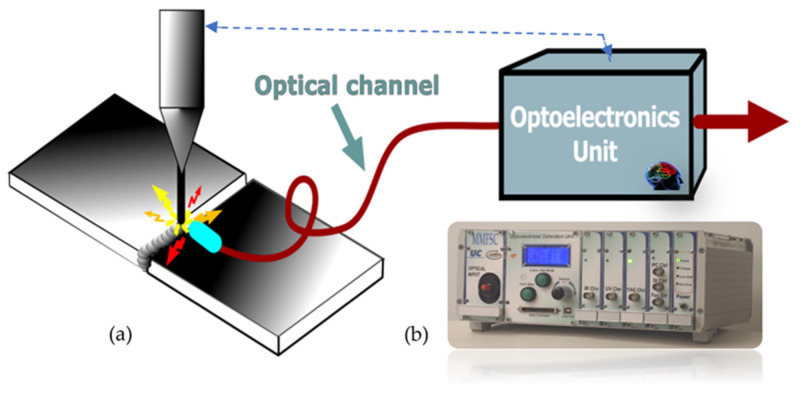
A general schematic illustration of a Smart Photonic Sensor for online welding monitoring. It is based on the spectrometry of the Light from plasmas of the welding pool and a significant level of signal processing. In the welding process of two materials using different sources of energy (laser, TIG, etc.), the borders of two materials are converted to a liquid state. During this process, plasma is created from the welding pool. Inside the spectra of the emitted Light (LM) are encoded information concerning the elements in the welding process at any time and on each welding position of the pool. After being detected and online spectra are determined by employing fast, complex, appropriate algorithms and Artificial Intelligence, welding defects can be determined online, and actuation signals can be supplied by the output of the optoelectronic unit. (**a**) General view of the SPS system; (**b**) a view of an optoelectronic unit for laser welding monitoring developed in the frame of the European MMFSC Project (GRD1-1999-10248). Made by the author based on material courtesy of the Photonic Engineering Group of UC.

The analysis of the Light emitted during welding processes such as arc or laser welding also allows a robust online welding monitoring. As shown in Figure 13, plasma is provoked in the welding pool during the welding process. The emitted Light is captured, and their spectra are obtained and analyzed via their continuum signal in the optoelectronic unit. As the emission lines are associated with the different species or elements contributing to the plasma, i.e., those forming the workpiece and the protection gas employed in their different ionization stages via the appropriate signal processing, information correlated with the welding quality is obtained [78]. Furthermore, utilizing artificial intelligence such as Fuzzy Logic, Artificial Neural Networks (ANNs), Hierarchical Temporal Memories, and others [79,80,81,82,83,84], different kinds of defects can be discriminated. Finally, actuation signals can be offered by the optoelectronic unit.

Plasma Spectroscopic Photonic Sensors are essential to online monitoring welding processes where quality standards are very demanding in a broad set of industrial sector applications. Examples include the manufacturing of components for nuclear power stations (i.e., the so-called tube-to-tube sheet welding process in Nuclear Steam Generators [85]), the automotive industry (i.e., laser welding of Usibor alloys such as 1500 tailor-welded blanks [86]), and the aeronautic sector (i.e., to laser welding of several alloys such as INCONEL 718’welding processes [87] and also to control the laser focus [88]), to mention only three examples.

#### 4.3.3. Laser Spectrometric Photonic Sensors for Material’s Elements Composition

**Figure 14:**
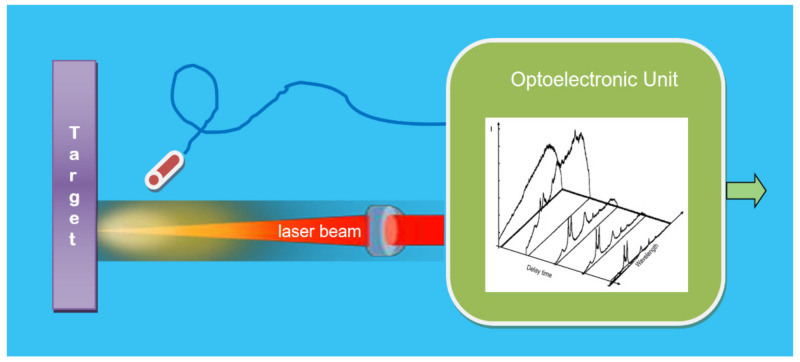
Schematic illustration of PS based on laser spectroscopy to detect and quantify chemical elements of materials (in solid, liquid or gas states). After interacting with the target, focused pulsed laser beams generate plasmas (including a stoichiometric ions concentration, Mx, of target material). The Light emitted by the plasma induced by the laser pulse is appropriately captured in an appropriate delay time (inaccurate synchronism with the laser pulse). Their spectra are obtained in the optoelectronic unit. After detection and properly processing, the information with the chemical elements concentration (and their quantification) is offered at the output of OU. Courtesy of the author.

By using Laser-Induced Breakdown Spectroscopy, LIBS, the composition of the elements of a given material (object or target) can be obtained [89,90]. As shown in Figure 14, when an appropriate laser pulse is focused onto the target sample, plasma integrated by ionized atoms and electrons is induced. The Light emitted by the plasma is captured in an adequate time (after the laser pulse of excitation), and then the spectrum is obtained. As every chemical element has its diagram level of energy, the acquired spectra have encoded the signatures of every ion inside the plasma. As the ions in the plasma provide a sample of the chemical composition inside the target, the kind of material can be identified from the spectra lines. Then, the amount in the sample was deducted from their intensity. Thus, by using PSs based on LIBS technique, the spectral signatures of chemical elements that constitute the target material in all states (solid, liquid and gas) can be obtained at the output of the optoelectronic unit.

PSs based on LIBS are useful for determining the elemental composition of solids, liquids and gases. It has the attraction that it is highly sensitive, fast, and non-contact. Therefore, PS-LIBS is appropriate to carry out elemental stoichiometric analysis of samples without destroying them, sensitively, quickly, and with minimal or no sample preparation. With the great advances of component technology and artificial intelligence, cost-effective miniaturized PS-LIBS are becoming increasingly popular. They have been implemented in a wide set of sectors applications, such as product and processes monitoring in Industry 4.0, environmental monitoring, medical diagnosis, biochemical-agent detection, and archaeological samples, just to mention a few [91,92,93]. Thus, PS-LIBS shows a great growth potential both in the scientific field and in various industries.

#### 4.3.4. Hybrid SPS for Physiological Reserve Diagnosis of Older People

It can be said that frailty and sarcopenia indicate the degree of loss of functional or physiological reserve and loss of skeletal muscle mass, respectively, that suggest or do not suggest vulnerability to adverse events during ageing. Thus, frailty could be defined as a multidimensional clinical entity that facilitates vulnerability to stressors due to the limitation of compensatory mechanisms. To estimate the functional physiological reserve, physicians, historically with a stopwatch in hand, have carried out consultations tests such as visually observing patients during Timed Up and Go and Chair tests as screening tools for geriatric fall risk assessment. More recently, these tests have been conducted in consultation by placing inertial sensors with accelerometers and gyroscopes on the patient, which provide more detailed information than the traditional method, but are intrusive and disturbing to the patients. On the other hand, many tests include a sitting/standing task, mainly in older people or in some types of rehabilitation, usually concerning the strength or movement of the lower body [94]. One of the most popular is the Timed Up and Go test, which involves getting up from a chair, walking 3–4 m, turning 180 degrees, coming back and sitting down again, and measuring the time taken. Another similar test consists of repeating the lifting of a chair, and there is a relationship between the two [95,96].

**Figure 15:**
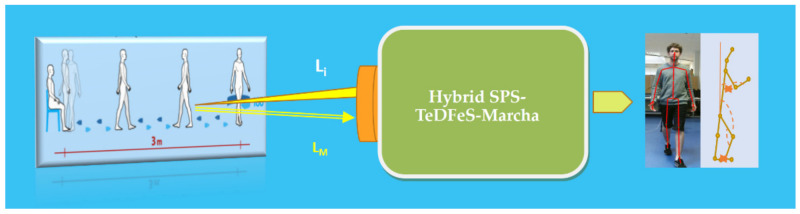
Schematic illustration of contactless hybrid SPS based on structured Light, flight time, and a smart chair. The patient is eliminated during the test with structured infrared Light (Li). The returning Light from the patient, LM, is then detected and adequately processed. This is collected simultaneously with the information from a smart chair, combined “harmoniously” in the control and processing unit of the SPS. The results emerge in real-time, are stored and collected in a report, and can be viewed as the patient executes an adequately designed exercise. Made by the author based on material courtesy of the Photonic Engineering Group of UC.

To facilitate the physicians’ tasks, a contactless hybrid SPS device called “TeDFeS-March” has been developed to contribute to early detection, prevention, and early interventions, key issues in the care of the elderly when assessing their physiological reserve (Figure 15).

The SPS-TeDFeS-March integrates two sub-sensor systems automatically controlled by an OU [97]. One subsystem illuminated with structured infrared Light (not visible to the human eye) the patient, capturing the light returns resulting from its interaction, combining them with RGB (visible) images of the mentioned patient. Previous data is collected simultaneously with the information from a smart chair (second subsystem), combined “harmoniously” in the control and processing unit. Thus, the results emerge in real-time, are stored and collected in a report, and can be viewed as the patient executes an adequately designed exercise. With this, the clinical specialist has the objective information and the required data, duly structured facilitators of diagnoses of the physical functionality of the elderly, making the process more effective and efficient.

Through the developed sensor technology, without any physical contact and in real-time, the patient undergoes the exercise called “get up, walk and sit down again,” which can be summarized as “get up and walk”. The SPS-TeDFeS-March “follows” and quantifies in detail, in addition to the times, other relevant biomechanical variables, after the test [4]. It provides objective data on gait symmetry, arm swing, elbow, and knee angles, the height of each ankle, the inclination of the spine concerning the vertical, the length and speed of the step and stride, the maximum height that each ankle reached, the force of different muscle groups exerted by the patient when standing up or, if dropped when sitting, the asymmetries of the extremities, and the acceleration when standing up, among others [98,99]. The technology is being field tested in several services of two hospitals of the SCS in Santander, Spain.

#### 4.3.5. Smart Image Photonic Sensors for Intraoperative Cancer Detection

It is very well known that cancer is one of the leading cause of death worldwide. The earlier cancer can be detected, the better the chance of a cure. On the other hand, the success of resection surgery, the oldest cancer treatment, highly depends on the ability to determine as accurately as possible the tumor margins to infer the lowest harm to the organ where the tumor originated but without leaving residual tumor. Patients undergoing repeat surgical procedures have significantly lower survival rates. Commonly, boundaries of the surgical cavity have been inspected visually by a surgeon using a surgical microscope, but small projections and filaments of the tumor may escape detection. Further, removed tissue may be sectioned and inspected by a pathologist to ensure that a rim of normal tissue has been removed along with the diseased tissue. This may be done intraoperatively using frozen sections and followed up with a microscopic evaluation of stained sections for tumor-specific features—but stained sections are typically not available until days after the surgery.

The apparent lack of reliably observed contrast between tumor and normal tissue during surgery (and after) is believed to be one of the major factors leading to incomplete resection of tumors. Thus, surgical guidance systems need to help surgeons visualize the tumor margins precisely and guide them in making complete tumor resections during the initial surgery.

**Figure 16:**
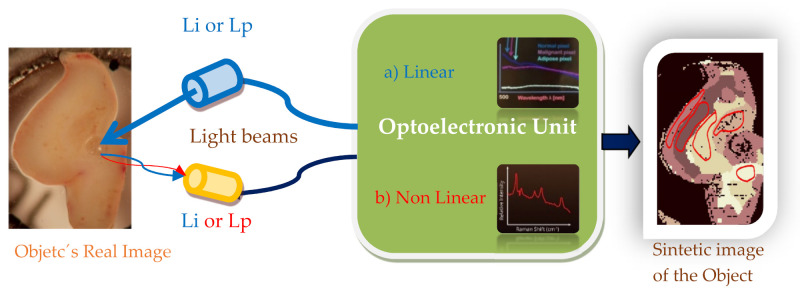
Illustration of a Smart Photonic Sensor able to perform, in quasi-real-time, the automatic discrimination of normal and tumor areas on the tissue of a human organ. The object is illuminated with a source of Light and after the interaction with the object: (**a**) By using linear scattering, a few accounts of Light’s photons (LM) of the same frequency return to the OU; the optical spectra are then obtained (inside the wavelength range of interest) and depending on the state of the tissue of the object, different spectra signatures are obtained for normal, diseased, and other types of tissues (if required); then, using the appropriate algorithms, each pixel is classified according to the type of tissue-state. In a basis of pixel per-pixel basis, a representative synthetic image of the state of the tissue is then reconstructed. (**b**) By using non-linear scattering, a very few accounts of Light’s photons (LM) of different frequency return to the OU in which their corresponding spectra are detected and correlated with the pixel state of the tissue; and (if required) a synthetic image (including the corresponding tissue type borders) can be reconstructed (and, if required, over-imposed on the real object’s image of the field of view of the surgeon). Made by the author based on material courtesy of the Photonic Engineering Group of UC.

By using the scattered lights from the tissues, BioPhotonic Sensors are very promising devices. Several sensor systems have been developed and successfully checked. Here, two cases are briefly presented: one based on linear scattering and the other on hybrid Raman non-linear scattering enhanced by plasmonic (SERS). An illustration of the block diagrams of both sensors is summarized in Figure 16.

Light scattering measurements are minimally invasive and allow the estimation of tissue state (healthy/disease) to guide the surgeon in resection surgeries. The scatter signatures can be captured on a per-pixel basis. Their spectra are obtained and analyzed using complex algorithms that conclude an actuation signal (nonmalignant or malignant or another type of tissue, adipose, for instance). According to the scatter parameters, the tissue is classified as normal organ cells or tumor cells. Tissue classification information for each spot of the plurality of spots is displayed. The classification information for each spot is portrayed as a pixel of an image, thereby portraying a map of identified tissue types. If required, the complete 2D image can be reconstructed, remarking the boundaries of each type of tissue. To help the surgeon make real-time decisions during surgery, the synthetized image can be over imposed onto the real image that his eyes see in his field of view of the organ/tissue he is working on.

Several approaches of Smart Photonic Sensors based on linear scattering have been reported and some patented [100]. In this international patent, a SPS is described for optically scanning a field of view, the field of view incorporating at least part of an organ exposed during surgery, and for identifying and classifying areas of the tumor within the field of view. The apparatus obtains a spectrum at each pixel of the field of view. The classification of pixels is performed by a K-Nearest-Neighbor type classifier (kNN-type classifier) previously trained on samples of tumors and organs classified by a pathologist. Embodiments using various statistical and textural parameters extracted from each pixel and neighboring pixels are disclosed. Results are displayed as a color-encoded map of tissue types to the surgeon.

On the other hand, relevant progress has been realized by using non-linear Raman scattering. This one was amplified in situ using plasmonic effects induced by previously doping the object’s tissue with appropriate nanoparticles. The current difficulty in visualizing the true extent of malignant brain tumors during surgical resection represents one of the primary reasons for the poor prognosis of brain tumor patients.

Surface-enhanced Raman scattering (SERS) has the potential to depict precisely the tumor extent with high sensitivity, specificity, and spatial resolution, and providing a promising platform to improve therapeutic efficiency.

A hand-held Raman scanner, guided by surface-enhanced Raman scattering (SERS) nanoparticles, to identify the microscopic tumor extent in a genetically engineered RCAS/tv-a glioblastoma mouse model has been reported [101]. This technology has a strong potential for clinical translation because it uses inert gold-silica SERS nanoparticles and a hand-held Raman scanner that can guide brain tumor resection in the operating room. This SPS device is more accurate than resection using white light visualization alone. This hand-held Raman probe (transducer) not only allowed near real-time scanning but also detected additional microscopic foci of cancer in the resection bed that were not seen on static SERS images and would otherwise have been missed.

Using the devices based on SERS spectroscopy for intraoperative image-guided resection is still a promising platform that requires more work [102,103,104].

Finally, it is worth recalling that the failure of complete tumor resection during cancer surgery is a leading cause of lethal recurrence and metastasis. Despite the very remarkable contributions on this specific subarea of SPS, achieving accurate delineation of tumor margins intraoperatively remains extremely difficult because the infiltrated nature of a tumor usually gives an obscure margin with spreading microtumors. More R&D efforts must be invested to reach a complete and entirely successful transference of these types of very required non-contact monitoring devices. Blanks are still left for further fulfillment.

#### 4.3.6. Toward BioPSs for Early Detection of Alzheimer through the Eye

There is no definite early diagnosis method for Alzheimer’s disease (AD), up to the best of our knowledge. It can only be confirmed postmortem through histopathological identification in the brain of its characteristic features, including beta-amyloid (Aβ) plaques. Thus, there is a clear need for a technique capable of improving the in-vivo diagnosis of AD at early stages.

While Amyloid PET has a high negative predictive value for discriminating AD from non-amyloid dementias, the positive predictive value remains low for application at an individual patient level. Therefore, Tau PET remains investigational at present. Further, the expense and radiation exposure from PET remain limitations for large-scale application of PET for routine screening purposes or application in developing countries.

**Figure 17:**
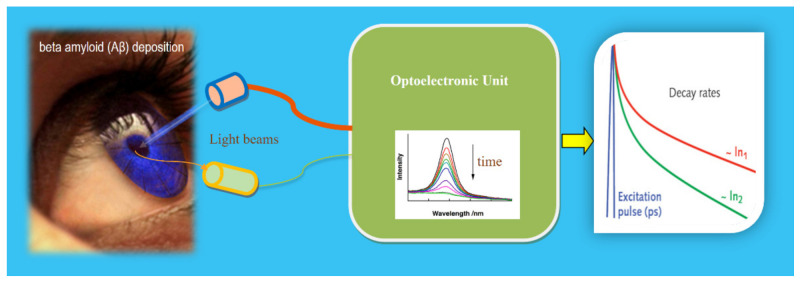
Illustration of a BioPhotonic Sensor (BioPS-FLES) for early detection of Alzheimer’s disease. The excitation laser pulsed light from the optoelectronic unit in the interaction with the lens of the eye provokes a fluorescent light (LM) that, after detection and adequate treatment in the OU, the output is given based on the decay times. The higher the beta-amyloid, the lower the fluorescent decay times [105]. Courtesy of the author.

The deposition of beta-amyloid (Aβ) on elements of animals and our eyes has been demonstrated [105,106]. Thus, as a unique window of the brain, clinical studies using the decay times of the fluorescent Light from a ligand in an element (lens, retina) differentiate between patients with probable AD and healthy volunteers (HV) as well as some new analyses [107]. The BioPS used (denominates as Fluorescent Ligand Eye Scanning, FLES) can be interpreted as shown in Figure 17. The BioPS-FLES used is a combination of a fluorescent ligand and a laser eye scanning device. The fluorescent ligand was dosed topically to the lower eyelid the evening before the measurement day. The BioPS-FLES system is a combination product of ligand-device that detects the fluorescent signature of the applied ligand bound to AD in the supranucleus of the lens [106].

The ligand, aftobetin hydrochloride, is an ophthalmic ointment that is applied topically to the eye. It measures the emitted fluorescence signal of aftobetin-HCl bound to Aβ aggregates in the supranuclear region of the lens. The BioPS can scan the supranucleus area of the lens quickly and provides a numerical output, which is referred to as Fluorescence Uptake Value (FUV). Contrary to other techniques, the device does not require an operator’s (expert) read or any analysis of images.

The clinical trials showed that AD and healthy volunteer groups could be differentiated with a sensitivity of 85% and specificity of 95% (*p* < 0.001).

In current studies, it has been demonstrated that retinal changes can reflect the pathology of the brain [108,109]. Thus, considering the understanding of retinal structures in AD, mild cognitive impairment (MCI), and preclinical AD, focusing on neurodegeneration and microvascular changes measured using optical coherence tomography (OCT) and optical coherence tomography angiography (OCTA) technologies, it is suggested that the impairment of retinal microvascular network and neural microstructure exists in AD, MCI, and even preclinical AD. Therefore, they can be used as potential biomarkers for early diagnosis of AD and monitoring of disease progression.

In the currents, the aim of uncovering biomarkers in the eye that may be used for early or preclinical detection of AD is possible but remains elusive. Research on the eye fluid, lens, and other ocular structures to detect early AD signs is promising, mainly because it is accessible during cataract surgery. However, while some early data has emerged about Aβ deposition in the lens and retina that are potentially unique to AD, there is some confounding data and disagreement. Similarly, non-invasive imaging with OCT has shown promise and disagreement about distinct, identifiable AD-related changes in the retina [110].

## 5. Conclusions

Sensors are the devices or systems developed to detect and capture measurands, in any domain and reproduce them in the electrical domain to be useful in real applications.

Photonics is a Key Enabling Technology or an Essential Technology for the development of science in Europe, the USA, and other leading nations worldwide.

The Photonics field or Science and Technology of Light, is understood as the set of techniques and scientific knowledge applied to the generation, propagation, control, amplification, detection, storage, and processing of signals of the optical spectrum, along with their technologies and derived uses.

Understood as any sensing approach that employs Light-based technologies, photonic sensors (a very relevant area of Photonics) experiment with strong socio-economic impacts in the first half of the 21st century. Therefore, it is expected that the vast Photonics and photonic sensor markets will experience a CAGRs of about 7% and 17%, respectively, between 2020 to 2025.

The optical transducer, optical channel, and optoelectronic unit are the three main parts of a photonic sensor that provide representative and faithful electrical signals of the measurand(s) on/in a given object or target. When equipped with some kind of intelligence (commonly in the OU) to provide actuation signals, the sensing device is transformed into a Smart Photonic Sensor.

Several sensor types can be defined according to the modulating technique used to encode the measurand, the interaction of Light-Object-Measurand, the way or mode to obtain the modulated Light, the measurand’s domain, the spatial distribution of the measurand in the object, the requirement to dope the object or not, and the technology used to build up the transducer.

In the current state of studies, a vast soup of terms, concepts, methods, effects, approaches, techniques, and technologies persist that seem to show different meanings which in reality are the same or are incorrectly used, inducing errors within the discipline.

A doctrinal conception of sensing using Light as an “umbrella” is herein offered, in which any sensing approach using Light Sciences and Technologies can be easily included. Below this “umbrella”, all mentioned terms, concepts, methods, techniques, technologies, sensing devices, etc., can be easily considered. With this proposal, any sensing approach using Light Sciences and Technologies will be quickly considered inside the sensing using Light area or, simply, photonic sensors, a key area of Photonics field.
